# Depression, Anxiety, and Post-Traumatic Stress Disorder Following Orthopedic War Injuries

**DOI:** 10.7759/cureus.13792

**Published:** 2021-03-09

**Authors:** Çağdaş Biçen, Mehmet Akdemir, Dilek Gülveren, Deniz Dirin, Ahmet Ekin

**Affiliations:** 1 Orthopedics and Traumatology, Izmir University of Economics Medicalpark Hospital, Izmir, TUR; 2 Orthopedics and Traumatology, Izmir Ekol Hospital, Izmir, TUR; 3 Clinical Psychology, Izmir University of Economics Medicalpark Hospital, Izmir, TUR; 4 Psychology, Izmir University of Economics Medicalpark Hospital, Izmir, TUR

**Keywords:** war injury, libya, depression, anxiety, ptsd

## Abstract

Introduction

There are ongoing wars worldwide, during which significant numbers of people are injured. Several studies have indicated that high rates of depression and anxiety are seen in war-injured patients.

Methods

Eighty-one male patients treated between November 2019 and January 2021 far from home in a Turkish hospital due to war injuries that happened in the Libyan Civil War were investigated. Demographic characteristics and injury data of the patients were evaluated regarding age, Injury Severity Score (ISS), location of injuries, type and mechanism of injuries, operations, and accompanying traumas. The psychological statuses of the patients were evaluated with the Beck Depression Inventory (BDI), Beck Anxiety Inventory (BAI), and post-traumatic stress disorder (PTSD) records filled out at the first admission to the hospital.

Results

The mean age of the patients was 29.8±7.7 (19-56) years. While 59 patients had fractures, 22 patients had only soft tissue injuries. Eighteen patients suffered from other accompanying injuries. While 85.2% of the patients showed symptoms of depression, 82.7% of the patients suffered from anxiety and PTSD symptoms were seen in 86.4% of the patients. Statistical analysis was performed to investigate the effects of injury severity, duration of hospitalization, number of operations, and age on depression, anxiety, and PTSD among these patients with war injuries. The results did not indicate any significant effect of injury severity, hospitalization duration, or operations.

Conclusion

Depression, anxiety, and PTSD are common in patients injured in wars. Injury severity does not seem to affect depression, anxiety, or PTSD in these patients.

## Introduction

Wars have affected humanity throughout history and continue to be ongoing worldwide. High rates of deaths and injuries have been reported [1]. Advances in military technology, the increased destructive power of weapons, and consequently the ensuing injuries are more devastating and mortality rates have increased in recent years. The majority of war traumas consist of musculoskeletal injuries [2]. When compared with daily orthopedic trauma practice, open fractures are reported at higher rates among war injuries due to explosives and gunshots [3]. A significant proportion of these patients need to be treated as polytrauma patients. Achieving patients’ preinjury functional capacities and management of the accompanying injuries are the main goals of treatment, but wars threaten also the psychology of the people. While managing the treatment of injuries, the psychological statuses of these patients are often disregarded. At the end of treatment, significant numbers of patients suffer from anxiety, depression, and post-traumatic stress disorder (PTSD). As a step of "whole patient care," psychiatric support must be organized for these patients [4].

The psychological conditions of victims of war have been investigated previously [5,6]. Studies were reporting psychological changes after brain injuries in wars [7,8]. In the literature studies addressing the psychological status of patients with orthopedic injuries, psychological difficulties were most often experienced by veterans or amputees [9,10]. These studies mostly reported on the professional support offered to these patients.

Victims of wars may not always have access to sufficient health support in their own countries; some of the victims must be transferred to neighboring or distant countries. In Libya, civil war arose in 2011 and is still ongoing and many people have been injured and have died. Our hospital has been involved in the treatment of patients injured in the Libyan Civil War since the beginning. We observed that a significant number of these patients suffered from psychological disorders. This study aimed to investigate the rates of depression, anxiety, and PTSD among patients who were injured during the Libyan Civil War and were receiving medical support far away from their home country. Another aim of this study was to observe the differences between the psychological statuses of patients of different ages and different trauma types and severities.

## Materials and methods

We retrospectively evaluated 103 patients who were treated in our hospital between November 2019 and January 2021 for injuries that occurred in the Libyan Civil War. The study group comprised patients with orthopedic injuries. All procedures were conducted in accordance with the ethical standards of the responsible committee (Institutional Ethical Approval No. B.30.2.İEÜSB.0.05.05-20-101). Data were obtained from the files of the patients and medical records of the hospital. The inclusion criteria were; first being injured in the Libyan Civil War, second having an orthopedic injury, and third being operated on. Patients under 18 years of age, patients with mental confusion, and patients whose medical records were lacking were excluded from the study. The demographic characteristics and injury data were evaluated regarding age, Injury Severity Score (ISS), injury location, injury type and mechanism, accompanying traumas, and operations. The ISS is a scoring system based on anatomical regions that are used for assessing trauma severity in patients with multiple injuries [11]. Scores of <9 represent mild injuries, 9-15 moderate, 16-24 severe, and ≥25 profound. We divided the patients into two groups regarding ISS. Patients with ISS values lower than 16 constituted the first group, having mild or moderate injuries, and patients with ISS values of 16 and above constituted the second group, having severe or profound injuries.

For psychological assessment, the Beck Depression Inventory (BDI), the Beck Anxiety Inventory (BAI), and the PTSD records filled out at first admission of the patients were evaluated. Twenty-two patients whose records of one or more of these three scales lacked in-patient files were excluded from the study.

The BDI was developed by Beck et al. in 1961 [12]. It consists of 21 items that are used to measure symptoms related to depression to evaluate emotional, cognitive, physical, and motor functions. Each item is scored between zero and three. The validity study of this inventory in the Arabic language was done by Rahat et al. in 2012 [13]. The BAI was developed by Beck for measuring anxiety levels in 1988 [14]. With 21 items scored between zero and three, it is a Likert-type assessment tool with which varying scores are given. To calculate the total score, the answers given to the items in the scale are summed. While the lowest score that can be obtained from the scale is zero, the highest score that can be obtained is 63. Higher total scores indicate an increase in anxiety severity. The validity of this inventory in the Arabic language was completed in 2000 by Al Nehar and Al Zubaidi [15].

The Clinician-Administered PTSD Scale (CAPS) was developed by Blake et al. for research and clinical follow-up. It contains a total of 25 questions [16]. Seventeen of the questions consider PTSD symptoms according to the DSM-III-R. The other eight questions consider accompanying symptoms of PTSD. The total score, obtained by totaling the frequency and severity scores of symptoms, gives an idea of ​​the severity of the disorder. The CAPS was prepared and developed in keeping with clinical needs.

Statistical analysis

Statistical evaluation was performed considering BDI, BAI, and PTSD scores together with ISS, age, accompanying trauma, fractures, and a total number of operations. SPSS 17 was used for statistical analysis. For assessment of categorical data, percentages, ratios, and averages were used. After dividing the patients into two groups (first group: ISS<16; second group: ISS≥16), the BDI, BAI, and PTSD scores were compared between the groups using the Mann-Whitney U test. Effects of accompanying traumas, presence of fracture, on BDI, BAI, and PTSD scores were also assessed with the Mann-Whitney U test. The effect of type of injury on the scores was evaluated using the Kruskal-Wallis test. Correlations of BDI, BAI, and PTSD scores with the age of the patients, duration of hospitalization, time from injury to admission to our hospital, and a number of operations were analyzed using Spearman’s correlation test. Values of p<0.05 were accepted as statistically significant.

## Results

A total of 81 patients were identified from the hospital’s database. At admission to our hospital, patients were moved to clinics according to types of injuries and necessary treatments. Patients with critical general statuses were directed to intensive care units. After the patients were taken to the clinics, the BDI, BAI, and PTSD scores were calculated by the department of psychiatry. The mean age of the patients was 29.8±7.7 (19-56) years and all patients were male. The mean time interval from injury to admission to our hospital was 8.8 months. Forty-three (53.1%) patients were injured by gunshots, 31 (38.3%) by explosives, and seven (8.6%) due to falling. While 59 (72.8%) patients had fractures, 22 (27.2%) patients had only soft tissue injuries. Eighteen (22.2%) of the patients suffered from accompanying injuries (Table 1).

**Table 1:** Demographics of the patients.

	n	%	Mean	Min-max	Mean SD
Age			29.8	19-56	7.7
Injury severity score			18	9-45	9.3
Injury type					
Fracture	59	72.8			
Soft tissue	22	27.2			
Injury mechanism					
Gunshot	43	53.1			
Explosive	31	38.3			
Fall	7	8.6			
Duration of hospitalization (day)			25.6	2-224	33.7
Accompanying injuries	18	22.3			
Number of operations			2.9	1-8	1.6

The average ISS value of the patients was 18±9.3 (9-45). The ISS was <16 in 40 patients (49.4%) and ≥16 in 41 patients (50.6%). Fractures were of the femur most commonly, for 20 patients, and of the tibia for 15 patients. Eight of the patients had fibula fractures accompanying tibia fractures. Nine had humerus fractures, eight had foot fractures, six had hand fractures, five had forearm fractures, four had vertebral fractures, two had acromion and clavicle fractures, and one had a patella fracture. Soft tissue injuries included nerve injury in 16 patients (including total and partial injuries; 10 sciatic, two peroneal, three radial, three brachial plexus, two ulnar, and two tibial nerve), tendon injury in five patients, ligament rupture in three patients, skin defect in two patients, and arterial injury in one patient. The average number of operations performed was 2.9 (1-8). Twenty-nine patients underwent plate-screw fixation, 10 patients intramedullary nailing, 10 patients K-wire fixation, three patients posterior segmental instrumentation, and one patient arthroplasty. External fixators were applied in 18 cases. Internal fixation was applied for 10 of these patients. Nine of them underwent plate-screw fixation and one had intramedullary nailing. Amputation was performed for seven patients. For 11 patients, foreign body excision was performed. Five patients underwent tendon repair and one patient had an arterial repair. Tendon transfers were applied for four patients and primary nerve repair for four patients. Skin grafting and flaps were performed for eight patients and two patients required fasciotomy. The mean hospitalization of the patients in our hospital was 25.6±33.7 (2-224) days.

According to the administered scales, only 12 (14.8%) patients showed no symptoms of depression. Eighteen (22.2%) of them had mild, 38 (46.9%) had moderate, and 13 (16.1%) had severe symptoms of depression. Fourteen (17.3%) patients presented without anxiety according to the BAI, while 43 (53%) patients presented with mild anxiety, 19 (23.5%) patients with moderate anxiety, and five (6.2%) patients with severe anxiety symptoms. Finally, 71 (87.7%) patients described symptoms of PTSD.

These scales were compared between patients with different trauma severities. Patients were divided into two groups, whereby the first group consisted of patients with ISS of <16 and the second group consisted of patients with ISS of ≥16. The BDI, BAI, and PTSD scores of the two groups were compared using the Mann-Whitney U test. There was no statistically significant difference between the groups (p=0.955, 0.906, 0.344; p>0.05). The effect of accompanying traumas on the scores of these 3 scales was also evaluated with the Mann-Whitney U test. A statistically significant difference was not observed in patients with accompanying injuries regarding BDI, BAI, or PTSD scores (p=0.937, 0.523, 0.686; p>0.05). Another comparative analysis according to injury type was performed again using the Mann-Whitney U test. Patients with fractures were compared with patients with only soft tissue injuries. There was no statistically significant difference between BDI, BAI, or PTSD scores (p=0.527, 0.533, 0.473; p>0.05) (Table 2). In the analysis of the relationship of BDI, BAI, and PTSD scores with patients’ ages and a number of operations, duration of hospitalization, and time from injury to admission to our hospital, only age was found to affect the BAI scores, but this difference was not statistically significant (p>0.05).

**Table 2:** Comparison of rates of depression, anxiety, and PTSD scores regarding injury severity, accompanying injuries, and the existence of fracture using Mann-Whitney U test. BAI: Beck Anxiety Inventory, BDI: Beck Depression Inventory, PTSD: post-traumatic stress disorder, ISS: Injury Severity Score Values of p<0.05 were accepted as statistically significant.

		BAI score (p-value)	BDI score (p-value)	PTSD score (p-value)
ISS score	<15	0.906	0.955	0.344
	≥16			
Accompanying injury	+	0.523	0.937	0.686
	-			
Fracture	+	0.533	0.527	0.473

## Discussion

Patients who are living in countries at war or in neighboring areas clearly present with higher depression and anxiety levels [17,18]. Severe injuries are seen at increased rates among war injuries. For those patients with long-lasting treatment or disability, both depression and anxiety are observed at higher levels. While planning treatment in order to achieve preinjury functional activities in these patients, the psychological statuses of the patients have to be considered and sufficient support has to be given.

During wars, health systems generally become insufficient in the affected countries. The injured may not always be treated in their own countries; they may be transferred to neighboring countries or areas overseas. The Libyan Civil War has been ongoing since 2011. Our clinic is one of the clinics that has taken action in treating patients injured in the Libyan Civil War since its early stages. We were able to improve the functional status of many patients, but we also observed that the patients were suffering from depression and anxiety. Studies are focusing on the management of the psychiatric treatment of Libyan society, but no specific studies have been conducted to date addressing the psychiatric conditions of the injured [19,20]. We thus aimed to evaluate the depression, anxiety, and PTSD levels of patients who were injured during the Libyan Civil War and treated in a distant country.

Depression has a range of occurrence of 3.2-19.8% in the general population while depression prevalence was reported as 42% after trauma in the literature [21]. In a review study, PTSD was shown to range from 17.5% to 42% in the first six months following injury [22]. When compared with the general population and with trauma groups, depression and stress rates were found to be significantly higher in our study group.

The effect of severity of injury on the psychology of the patients was examined in the literature previously. Williams et al. evaluated in their study; the relation of injury severity with symptoms of depression and PTSD [23]. The severity of the injuries ranged from tendon ruptures to mangled extremities and amputations. They revealed that 32% of their patients showed symptoms of PTSD and 20% of the patients had depression and injury severity didn’t affect the rates. In the study of Wiseman et al., depression, anxiety, and stress were evaluated in traumatically injured patients during hospital admission and at the third and sixth months [24]. The total rates of depression, anxiety, and stress were reported as 58.7% at the time of hospitalization and as 40.2% at the third month, and 23.9% at the sixth month after injury. They reported that depression, anxiety, and stress levels had no specific relation with ISS. When compared with both studies; the symptoms related to depression, anxiety, and PTSD among patients were thus apparently higher in our study. The physical functionality subscales of the patients support the findings of our study since no significant difference was found regarding injury severity for anxiety, depression, or PTSD symptoms. Patients with war injuries seem to have more psychological disorders when compared with the general trauma population.

One of the largest series investigating psychological disorders of veterans was published by Engelbrecht et al. [25]. They observed 500 veterans of the Second World War in the UK. The psychological symptoms including depression, anxiety, avoidance of social contact, and difficulty completing tasks were recorded. They reported that anxiety persisted in 38.7% of the patients and depression persisted in 34.5% of the patients in the first decade following the war. In the following three decades the anxiety symptoms were available in 13.9% of the patients and depression symptoms were available in 14.3% of the patients. In another study conducted by Jones et al., experiences of a sample of 225 UK army veterans between 1945 and 2000 were reported [26]. 20 themes were inquired related to, anxiety, depression, ability to keep employment, and illness attributed to combat exposure. The symptoms were mentioned between 53% and 86% of the veterans. They also found in this study that mentioned disorders were reported at higher rates at the earlier periods. In our study patients were observed in relatively short- term after injury, and depression and anxiety symptoms were available in rates of 85.2% and 82.7%.

In 2018, Tennent et al. performed a study on the psychological status of US military personnel who had traumatic lower extremity amputations [27]. They collected data of patient-reported outcomes like our study. Mental health disability of transfemoral and through knee amputee populations was compared. They recorded that mental disability was available in groups with 77% and 96% consecutively. Most common diagnoses were anxiety, depression, PTSD, and traumatic brain injury (TBI). The increased rates of anxiety, depression, and PTSD of our patient group were also similar to the rates mentioned in this study.

In the retrospective study of Melcer et al. short-term mental outcomes of military personnel who had severe extremity injuries during the Iraq and Afghanistan wars were investigated [28]. They compared outcomes of amputee and non-amputee patients. The mean of ISS was 15.6 in amputee and was 15.3 in non-amputee patients. Mental health diagnosis and utilization of psychiatric clinics were recorded higher among amputees. But when compared with non-amputees; amputees showed up with reduced PTSD by approximately 50%. Though ISS, non-healing wounds, osteomyelitis, and complication rates were similar between groups, the reason for higher rates of PTSD among non-amputees has not been clarified. The mean of ISS of our patients was 18; 91.4% of our patients were non-amputees and were heavily injured. PTSD was present in 87.7% of our patients. We observed that type of injury and number of operations underwent didn’t affect rates of PTSD in our patient group.

In the study performed by Asadollahi et al., psychological disorders of patients who were heavily injured due to landmines in a province of Iran neighboring to Iraq border were investigated [29]. When compared with their general population anxiety, depression and PTSD was found very high in the patient injured by landmines. Depression was present in 79.6% of the victims and anxiety was present in 77.4% of the patients. Though these patients could not achieve adequate medical, social, and economic support; psychological recovery remained insufficient. Absence of rehabilitation after trauma, level of education, and environmental factors were accused of these higher rates

Twamley et al. conducted a study of veterans with a history of traumatic brain injury [30]. They divided their patients into two groups based on whether they were facing social difficulties. A diagnosis of depression was present in 52.7% of the patients who did not have social difficulties and 78.1% of those who did face social difficulties. PTSD was diagnosed in 66.90% and 87.50%, respectively. The increased rates of signs of depression and PTSD in our patients were as high as those seen in patients with TBI.

There are some limitations to our study. First, we had a limited number of patients. The second limitation was the design of the study. We designed our study retrospectively. Since the patients had come from another country, it would have been difficult to design a prospective study and evaluate the changes in a longer-term follow-up of the patients. The patients in this study were all male. Finally, the scales that we used for evaluating the psychological statuses of the patients were self-reported scales. Consequently, we can not accept the results as diagnoses.

## Conclusions

War injured is suffering from psychological disorders. Patients who are in the acute phase of their injuries; might have had feelings of distrust in the future and fear of death. But it is observed that patients show up with psychological disorders also in longer periods following injury. Psychology is thought to affect acute and long-term recovery in cases of war injuries, and also has an impact on functional gain and quality of life. The results of our study show that war-injured individuals have dramatically increased depression, anxiety, and PTSD symptoms. Severity and type of injury, age, length of hospitalization, or a number of operations underwent don’t have a significant effect on the symptoms.
